# The ‘reverse loop’ technique for difficult left ventricular access during percutaneous balloon mitral valvuloplasty

**DOI:** 10.1093/ehjcr/ytaf457

**Published:** 2025-09-18

**Authors:** Milind S Phadke, Saurabh V Limaye, Pratap J Nathani

**Affiliations:** Department of Cardiology, Lokmanya Tilak Municipal General Hospital and Medical College, Sion, Mumbai 400022, India; Department of Cardiology, Lokmanya Tilak Municipal General Hospital and Medical College, Sion, Mumbai 400022, India; Department of Cardiology, Lokmanya Tilak Municipal General Hospital and Medical College, Sion, Mumbai 400022, India

**Keywords:** Mitral stenosis, Percutaneous balloon mitral valvuloplasty, Reverse loop technique

## Case description

**Figure 1:**
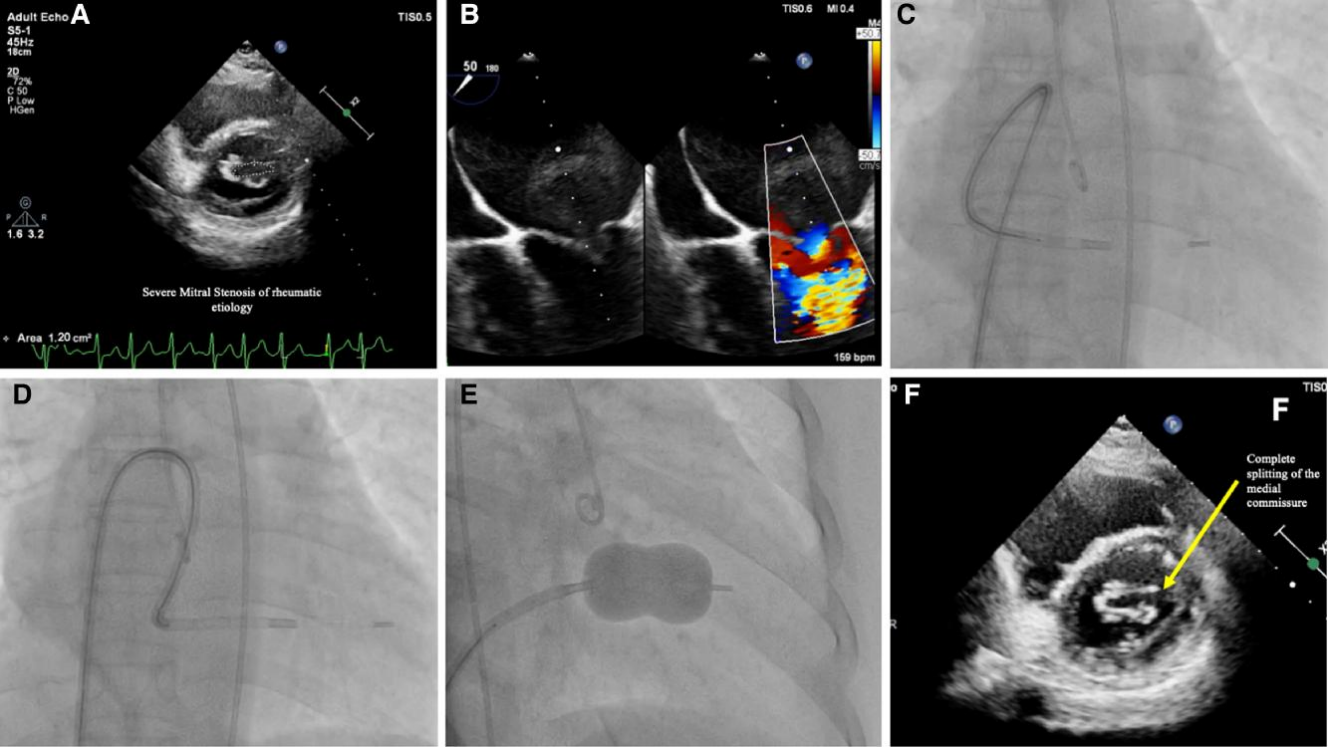
(*A*) Transthoracic echocardiography suggestive of severe mitral stenosis of rheumatic aetiology. (*B*) Transoesophageal echocardiography suggestive of mitral stenosis with hugely dilated left atrium. (*C*) Reverse (alpha) loop of balloon delivery catheter in left atrium. (*D*) Entry in the left ventricle across stenotic mitral valve. (*E*) Balloon inflation across mitral valve with relief of the lateral commissure. (*F*) Post-percutaneous balloon mitral valvuloplasty increase in mitral valve area with complete splitting of the medial commissure.

A 46-year-old woman with severe rheumatic mitral stenosis (mitral valve area 1.2 cm², mean gradient 10 mmHg) and a markedly dilated left atrium (LA) was scheduled for percutaneous balloon mitral valvuloplasty (PBMV) (*Figure 1A and B*). Transoesophageal echocardiography excluded left atrial appendage thrombus (see Supplementary material online, *Video S1*).

Percutaneous balloon mitral valvuloplasty was planned using a 26 mm Accura balloon (Vascular Concepts). Following successful transseptal puncture, a spring guide wire (Suretech Medical Inc.) was positioned in the LA. The mean left atrial pressure was 16 mmHg. Multiple attempts to enter the LV using the standard entry stylet were unsuccessful due to the markedly dilated LA (see Supplementary material online, *Video S2*). Ultimately, a ‘reverse loop’ was formed using the balloon catheter in the LA (see Supplementary material online, *Video S3*).

The stylet was rotated counterclockwise to form a loop within the dilated LA as opposed to the usual clockwise torque. It was then withdrawn approximately 8–10 cm to allow the floppy tip of the balloon catheter to float downward towards the mitral valve and enter the LV (*Figure 1C*). The balloon thus could cross the mitral valve after directing to LV apex (*Figure 1D*). Inflation was then performed in usual manner (*Figure 1E*; Supplementary material online, *Video S4*). Post-PBMV, the mitral valve area increased to 1.7 cm² with mild Mitral Regurgitation (MR), complete commissural splitting, and a reduction in mean left atrial pressure to 9 mmHg.

The reverse loop technique is particularly helpful in cases with a low septal puncture site or significantly dilated LA. Care must be taken to avoid entrapment of the balloon among chordae tendineae, which could worsen MR. Ensuring that the partially inflated balloon reaches the LV apex before final inflation reduces this risk.^1–3^

## Supplementary Material

ytaf457_Supplementary_Data

## Data Availability

The data underlying this article will be shared on reasonable request to the corresponding author.

## Notes

**Consent:** The authors affirm that the patient has given their written informed consent for the publication of this case report, including the associated images and supplementary videos.
